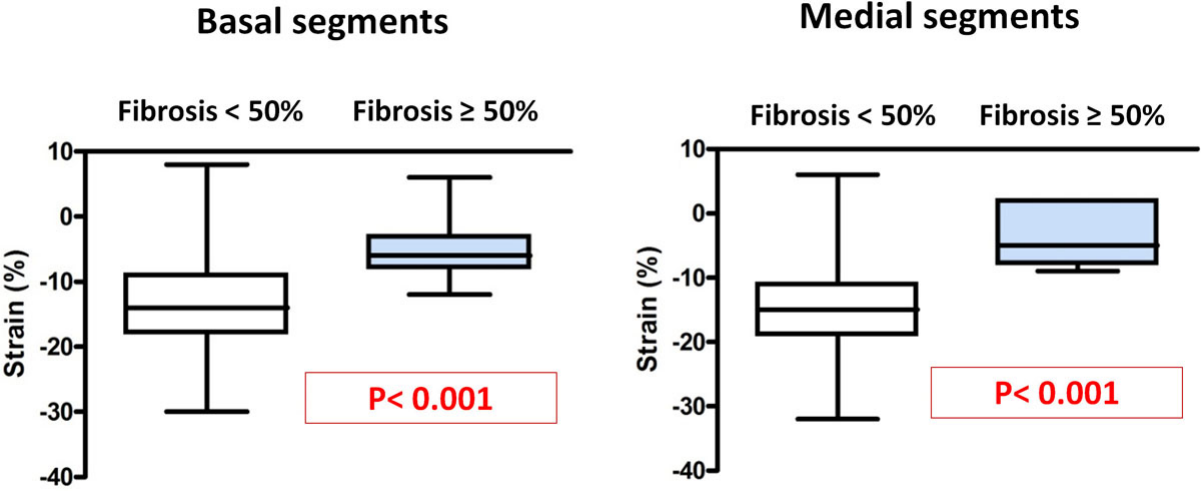# Hypertrophic cardiomyopathy and fibrosis: correlation between late gadolinium enhancement on CMR and speckle tracking imaging using Ultrasound

**DOI:** 10.1186/1532-429X-17-S1-P307

**Published:** 2015-02-03

**Authors:** Jennifer Cautela, Alain Lalande, Gilbert Habib, Franck Thuny, Alexis Jacquier

**Affiliations:** 1 Cardiologie, Assistance Publique Hôpitaux de Marseille, Marseille, France; 2 LE2I, UMR CNRS 6306, Université de Bourgogne, Faculté de Médecine, Dijon, France; 3 Radiologie, Assistance Publique Hôpitaux de Marseille, Marseille, France

## Background

Hypertrophic cardiomyopathy (HCM) is the most frequent genetic cardiovascular disorder and represents one of the most common cause of heart related sudden death in young adults. Myocardial fibrosis seems to be an independant predictor of adverse events including sudden death, ventricular arrhythmias and heart failure. While late gadolinium enhancement (LGE) on Cardiac Magnetic Resonance (CMR) is actually the gold-standard to detect fibrosis, new techniques are being evaluated such as 2D strain echocardiography.

## Purpose

To assess the relationship among segmental myocardial fibrosis, detect and quantified on CMR and segmental longitudinal strain (SLS) and to determine a threshold of SLS suggesting a significant myocardial fibrosis.

## Methods

A prospective, single-centre, observational study including all patients with HCM from 01/2012 to 01/2013. 1,5T CMR were performed in all patients with cine sequences (4, 2 chambers, Lvot, and short axis using standard parameters) and late gadolinium enhancement (LGE) (3D IR Flash, 10 to 15min after 0.2mmol/kg). Speckle tracking imaging echocardiography was performed on all the patients. Extent of LGE was determined semi-automatically by a dedicated software. The analysis was conducted on median and basal myocardial regions (to match the CMR and STI analysis, apical segments were excluded).

## Results

72 patients were prospectively included (mean 51.6 ± 15.8 years, 76.4% males) and 41 (57%) with LGE. Patients with fibrosis have higher wall thickness (22,1 ± 6,2mm vs 16,8 ± 3,3mm, p<0,001). There was a significant correlation between segmental STI and LGE in the basal level (r = 0.26, p <0.001) and mid level (r = 0.32, p <0.001). This correlation between segmental STI and LGE was independent of myocardial thickness (p<0.001). The threshold of segmental STI was -12.5% for the basal area (Se = 100%, Sp = 57%) and 9% for the mid level (SE = 100%, Sp = 83%) to differentiate segments with part of fibrosis ≥ 50%.

## Conclusions

Myocardial fibrosis is significantly associated with depressed segmental longitudinal strain in patients with HCM and will be useful to detect non-invasely important segmental fibrosis.

## Funding

None.

**Figure 1:**